# Characterisation of the Mesial Root and Middle Mesial Canal Morphology in Mandibular First Molars: An Ex Vivo Micro‐CT Study

**DOI:** 10.1111/aej.70056

**Published:** 2026-01-09

**Authors:** Nuno Rodrigues dos Santos, Jorge N. R. Martins, Mário Rito Pereira, António Ginjeira

**Affiliations:** ^1^ Faculdade de Medicina Dentária Universidade de Lisboa Lisboa Portugal; ^2^ Grupo de Investigação em Bioquimica e Biologia Oral, Unidade de Investigação em Ciências Orais e Biomédicas (UICOB), Faculdade de Medicina Dentária Universidade de Lisboa Lisboa Portugal; ^3^ Centro de Estudo de Medicina Dentária Baseada na Evidência (CEMDBE) ‐ Cochrane Portugal, Faculdade de Medicina Dentária Universidade de Lisboa Lisboa Portugal; ^4^ LIBPhys‐FCT UID/FIS/04559/2013, Faculdade de Medicina Dentária Universidade de Lisboa Lisboa Portugal; ^5^ Egas Moniz Center for Interdisciplinary Research (CiiEM) Egas Moniz School of Health & Science Almada Portugal

**Keywords:** endodontics, mandibular molars, micro‐CT, middle mesial canal, root canal anatomy

## Abstract

This ex vivo study characterised the mesial root morphology of mandibular first molars with a middle mesial canal (MMC) using micro‐CT. Among 469 scanned molars, 42 (8.96%) presented an MMC. Morphological parameters analysed included canal configuration, chamber orifice features, dentine thickness, isthmus type, and aspect ratio. Twenty‐four configurations were found, with the confluent type most prevalent (90.48%). Three separate orifices occurred in 64.29% of teeth, and MMCs were mainly in the coronal and middle thirds. Fully independent MMCs were rare (2.38%). Mean dentine thickness 2 mm below the furcation was 1.31 mm (MB), 1.03 mm (MMC) and 1.28 mm (ML). The mean aspect ratio was 2.90. Accessory canals appeared in 28.58%, and an independent MMC foramen in 38.09%, mainly on the mesial wall. Type III isthmus predominated (52.38%). Mesial roots with MMCs exhibit marked anatomical variability, with thin dentine around the MMC requiring careful endodontic management.

## 1 Introduction

The primary objective of endodontic treatment is to prevent or treat apical periodontitis. Achieving this objective depends on effective chemomechanical preparation and three‐dimensional obturation of the entire root canal system, processes that are directly influenced by the clinician's knowledge of internal tooth morphology [1, 2].

The mesial root of mandibular molars exhibits a highly complex internal anatomy, including multiple canals, isthmuses, and apical deltas. This anatomical complexity has been associated with increased technical difficulty during treatment and lower success rates when compared with less complex root systems [2, 3]. The mesial root typically contains two canals: the mesio‐buccal canal (MBC) and the mesio‐lingual canal (MLC). However, studies indicate that the internal and external anatomy of mandibular molars is highly variable. Numerous authors have reported the presence of an additional main canal in the mesial root, known as the middle mesial canal (MMC) [4, 5]. Failure to identify and treat additional canals such as the MMC has been associated with persistent infection and unfavorable treatment outcomes, underscoring the clinical relevance of accurately characterising this anatomical variation [6, 7].

Several techniques have been employed to study the root canal morphology of mandibular molars, relying on two‐dimensional evaluation methods such as radiography, cross‐sectioning, scanning electron microscopy and stereomicroscopy [8]. Although informative, these approaches are limited in their ability to capture the three‐dimensional complexity of the root canal system. In recent years, micro‐computed tomography (micro‐CT) imaging has become increasingly important in the study of root canal system morphology. Its capacity to provide high‐resolution, three‐dimensional, nondestructive imaging has established micro‐CT as a reference method for detailed anatomical assessment. It offers a detailed visualisation of tooth morphology and has proven to be an invaluable tool for both researchers and practitioners [9, 10]. Although the anatomy of the mesial root of mandibular first molars has been extensively studied, much of the literature relies on former research methods that may present limitations, such as methodological divergencies or technical challenges while exploring root canal anatomy [3, 11, 12]. Consequently, comprehensive data describing the full morphological features of the mesial root specifically in mandibular first molars with an MMC remain limited. Therefore, this study aimed to fully characterise the morphological features of the mesial root of mandibular first molars with an MMC using micro‐CT imaging.

## 2 Material and Methods

### 2.1 Sample Selection and Scanning Protocol

A total of 469 mandibular first molars were obtained from a local tooth bank, following prior approval by the local ethics committee (registration number CE‐FMDUL202418). Inclusion criteria comprised mandibular first molars with two separate roots, fully formed apices, and absence of previous endodontic treatment. Exclusion criteria included presence of restorations extending into the pulp chamber, caries affecting the pulp chamber or mesial root, external or internal root resorption, visible root fractures or cracks, severe calcifications preventing canal identification, and developmental anomalies. All teeth meeting the inclusion/exclusion criteria were selected. Information on patient age and gender was not available, as the teeth were extracted for purposes unrelated to this research and preserved in a 0.5% chloramine solution after the removal of soft tissue residues. Each tooth was mounted in a specimen holder and subjected to micro‐CT to examine the internal structure of the mesial root, using a SkyScan 1275 scanner (Bruker, Kontich, Belgium). The scanning parameters were set to a pixel size of 19.61 μm, with an x‐ray tube voltage of 80 kV, x‐ray intensity of 125 μA, a rotation step of 0.5°, full 360° rotation, and an image resolution of 1944 × 1536 pixels. The captured projection images were reconstructed using NRecon v1.7.3.1 software (Bruker), and DataViewer v1.4.4 (Bruker) was employed to examine the root and canal system morphology in multiple perspectives. Furthermore, 3D models were generated by segmenting the dentine and canal system using CTAn (v1.16, Bruker) and CTVol (v2.3, Bruker) software. Of the original 469 M, 42 specimens, representing 8.96%, were identified as having a MMC, according to the classification provided by Versiani et al. [5] (see below), and were subjected to further analysis of major and apical anatomy. All morphological assessments were performed by one observer (N.R.S.), and the observations were subsequently validated by two additional evaluators (J.N.R.M. and M.R.P.). All three observers had been previously calibrated according to the criteria described by Versiani et al. [5].

### 2.2 Mesial Root and Middle‐Mesial Canal Major Characteristics

The morphology of the mesial root canal configuration was analysed according to Vertucci's classification [2] and was supplemented with additional configurations. The middle‐mesial root canal configuration was classified according to Versiani et al. [5] into:

- Independent: three independent canals from pulp chamber to apex;
- Fin: in the coronal third, the MMC orifice is linked to the MBC and/or MLC orifices by a groove, yet all mesial canals exit the root through three separate foramina;
- Confluent: the MMC exits the pulp chamber, either independently or connected to the other mesial canals, and merges with the MBC and/or MLC through transverse anastomosis, intercanal connections or isthmus along its path to the apical foramen.

Additionally, the following characteristics of the middle mesial root canal configuration were also recorded: the type of chamber orifice (whether independent or confluent with the MBC and/or MLC); its position within the root (coronal, middle and/or apical); and its anatomical configuration (confluent with the MBC and/or MLC, or independent). The minimum periradicular dentine thickness (the smallest distance between the inner canal surface and the external root surface in any direction) was also measured in millimetres (mm) for each root canal at the 2 mm axial level below the furcation area (representing the periradicular area), using the measurement tool in the software (DataViewer v1.4.4, Bruker).

### 2.3 Mesial Root and Mesial Root Canals Apical Anatomy

Regarding the mesial root apical anatomy, the following parameters were determined and recorded: the aspect ratio of each mesial root calculated at the 3‐mm axial level coronal to the anatomic apex (representing surgical root resection level) (by dividing the root's maximum cross‐sectional diameter [*D*_max_] by its minimum diameter [*D*_min_]). In this context, Dmax represents the distance between the two most distant pixels on the two‐dimensional (2D) slice, while Dmin is defined as the longest chord drawn perpendicular to *D*_max_; the number of accessory canals and apical foramina; the presence of a middle mesial independent foramen (yes/no) and its position (mesial or distal root wall); the isthmus type (classified according to Hsu and Kim [13]) (Type I: two or three separate canals are present without any visible communication between them; Type II: two canals are present with a connection between the main canals; Type III: similar to Type II, but with three canals instead of two; Type IV: the canals extend into the isthmus region; and Type V: characterised by a continuous connection or corridor across the entire section); and the vertical distance from the main foramen to the anatomic apex (distance, measured in millimetres, between a horizontal plane intersecting the anatomic apex and another horizontal plane intersecting the centre of the foramen of each canal, while being parallel to the long axis of the root).

### 2.4 Statistical Analysis

All data were analysed using descriptive methods. Continuous variables were summarised using mean and range, while categorical variables were expressed as percentages. Rounding was standardised to two decimal places for continuous measures and to whole numbers for counts. No inferential statistical tests were performed, as the aim of the study was solely to provide a detailed morphological description of mandibular first molars with a MMC, rather than to compare groups or test hypotheses. This approach is consistent with the exploratory and descriptive nature of micro‐CT anatomical investigations.

## 3 Results

### 3.1 Mesial Root and Middle‐Mesial Canal Major Characteristics

**FIGURE 1:**
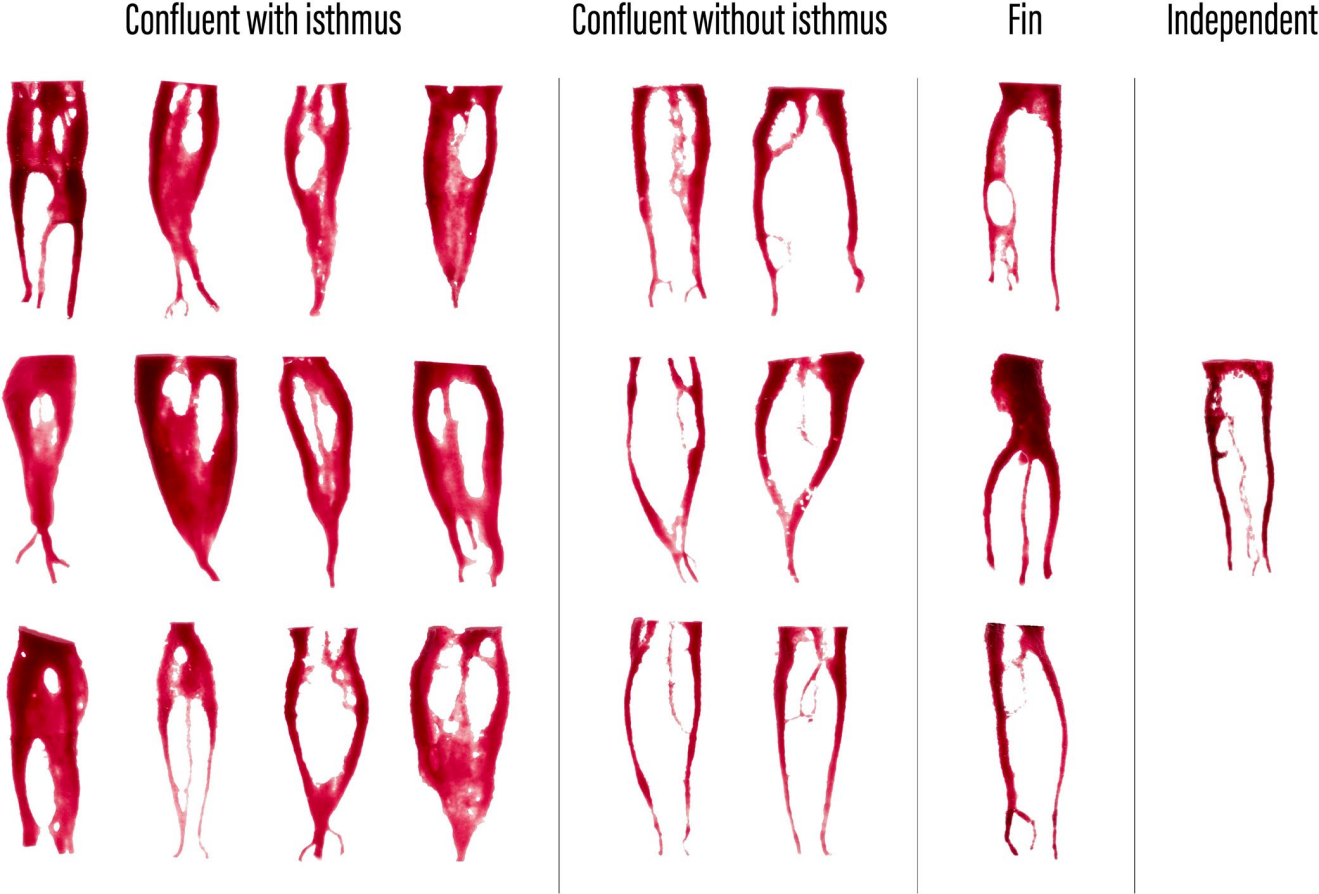
Representative images of the anatomical variations of the mesial root, showing different types of middle mesial canal configurations.

The mesial root exhibited significant anatomical variation (Figure 1), with the root canal system being classified into 24 different configurations. The most common morphologies were the complementary root canal configurations 3‐2‐1 (*n* = 7; 18.87%), followed by 2‐3‐2‐1 (*n* = 4; 9.52%). Additionally, confluent anatomy was the most prevalent anatomical variation, accounting for 90.48% (*n* = 38) of the sample. Overall, the majority of specimens had three independent pulp chamber orifices (*n* = 27; 64.29%), and the MMC was most commonly observed in the coronal and middle thirds of the root. A fully independent MMC extending along the entire root length was identified in only 1 root (2.38%). At the 2 mm axial level below the furcation, the average periradicular dentine thickness was 1.31 mm for the MBC (range: 0.71–1.91 mm), 1.03 mm for the MMC (range: 0.68–1.40 mm), and 1.28 mm for the MLC (range: 0.78–1.82 mm). Table 1 summarises the key characteristics of the mesial root and MMC.

**TABLE 1:** Mesial root and mesial root canals major characteristics.

Sample	Mesial root canal configuration	Middle‐mesial root canal configuration				Periradicular minimal dentine thickness (mm)^a^		
		Configuration type	Chamber orifice	Root position	Anatomy	Mesiobuccal	Middle‐mesial	Mesiolingual
1	2‐3‐2‐1	Confluent with isthmus	CONF‐ML	Middle	CONF‐ML	1.33	0.94	1.13
2	3‐4‐2‐4	Confluent without isthmus	IND	Coronal and Apical	CONF MB	0.90	0.94	1.41
3	3‐2‐3	Confluent with isthmus	IND	Coronal and apical	CONF‐MB	1.12	0.94	1.14
4	2‐1‐2‐3	Confluent with isthmus	CONF‐ML	Apical	CONF‐MB	1.06	—^b^	1.14
5	2‐3‐2	Confluent with isthmus	CONF‐MB	Middle	CONF‐ML	1.02	1.06	1.14
6	3‐2‐1‐3	Confluent with isthmus	IND	Coronal and apical	CONF‐MB	1.53	—^b^	1.41
7	3‐1	Confluent with isthmus	IND	Coronal	CONF‐MB AND ML	1.29	—^b^	1.22
8	3‐2‐1	Confluent with isthmus	IND	Coronal	CONF‐ML	1.18	—^b^	1.33
9	1‐3‐1‐3	Confluent with isthmus	CONF‐MB AND ML	Middle and apical	CONF‐ML	1.09	0.90	0.78
10	3‐4‐3‐2	Confluent with isthmus	IND	Coronal and middle	CONF‐MB	1.18	0.86	1.09
11	3‐2‐3‐2	Confluent without isthmus	IND	Coronal and middle	CONF‐MB	1.37	0.98	1.09
12	3‐4‐3‐2‐1	Confluent without isthmus	IND	Coronal and middle	CONF‐ML	1.18	0.90	1.14
13	3‐2‐1‐3	Confluent with isthmus	IND	Coronal and apical	CONF‐ML	1.38	0.98	1.22
14	1‐3	Fin	CONF‐ML	Middle and apical	CONF‐ML	0.71	0.90	0.82
15	2‐3‐2‐1	Confluent with isthmus	CONF‐MB	Middle	CONF‐MB	1.38	—^b^	1.14
16	3‐2‐1	Confluent with isthmus	IND	Coronal	CONF‐ML	1.09	—^b^	1.13
17	3‐4‐5‐3‐2	Confluent with isthmus	IND	Coronal and middle	CONF‐ML	1.29	1.02	1.53
18	2‐3‐2‐1‐3‐1‐2‐3	Confluent with isthmus	CONF‐MB	Coronal	CONF‐MB	1.29	1.29	1.41
19	3‐3	Independent	IND	All	IND	1.33	1.26	1.45
20	2‐3‐4	Fin	CONF‐MB	Middle and apical	CONF‐MB	1.37	—^b^	1.49
21	2‐3–2‐3	Confluent without isthmus	CONF‐MB	Coronal and apical	CONF‐ML	1.61	1.09	1.25
22	3‐1‐3	Confluent with isthmus	IND	Coronal and apical	CONF‐MB	1.28	1.10	1.25
23	3‐2	Confluent without isthmus	IND	Coronal and middle	CONF‐ML	1.55	0.96	1.31
24	3‐2‐3	Confluent without isthmus	IND	Coronal and apical	CONF‐ML	1.41	0.92	1.29
25	3‐2‐3	Confluent without isthmus	IND	Coronal and apical	CONF‐ML	1.22	0.78	1.29
26	3‐2‐1	Confluent without isthmus	CONF‐ML	Coronal	CONF‐ML	1.57	—	1.45
27	2‐3‐2	Confluent without isthmus	CONF‐ML	Middle	CONF‐ML	1.14	0.90	1.09
28	3‐2‐3‐2‐3	Confluent without isthmus	IND	Coronal and apical	CONF‐MB	1.56	1.28	1.51
29	2‐3‐2	Confluent without isthmus	CONF‐MB	Middle	CONF‐MB	1.03	0.68	0.99
30	3‐2‐3‐2‐1	Confluent without isthmus	IND	Coronal and middle	CONF‐MB AND ML	1.34	1.36	1.34
31	3‐2‐1	Confluent with isthmus	IND	Coronal	CONF‐ML	1.27	1.07	1.29
32	3‐2‐1	Confluent with isthmus	IND	Coronal	CONF‐MB	0.99	0.70	0.99
33	3‐1‐2‐4	Confluent with isthmus	IND	Coronal an apical	CONF‐MB	1.84	1.36	1.54
34	2‐3‐2‐1	Confluent with isthmus	CONF‐MB	Coronal and Middle	CONF‐MB	1.41	1.27	1.23
35	3‐4‐3‐2	Confluent without isthmus	IND	Coronal and middle	CONF‐ML	1.27	0.88	1.43
36	3‐2‐3‐2	Confluent without isthmus	IND	Coronal and middle	CONF‐ML	1.56	—^b^	1.62
37	3‐2‐1	Confluent without isthmus	CONF‐MB	Coronal	CONF‐MB	1.58	1.21	1.49
38	4‐3‐2	Confluent without isthmus	IND	Coronal and Middle	CONF‐ML and MB	1.91	1.40	1.82
39	3‐2‐1	Confluent with isthmus	IND	Coronal	CONF‐ML	1.18	1.07	1.47
40	2‐3‐2‐3	Fin	CONF‐ML	Middle and apical	CONF‐ML	1.36	0.94	1.65
41	3‐2‐3	Confluent with isthmus	IND	Coronal and apical	CONF.MB	1.41	0.90	1.18
42	2‐3‐2‐1	Confluent with isthmus	IND	Coronal	CONF‐ML	1.12	1.01	1.14

Abbreviations: CONF, confluent; IND, independent; MB, mesiobuccal; ML, mesiolingual; mm, millimetres.

^a^ Measured 2‐mm below the root furcation area.

^b^ Represent cases at which no middle‐mesial root canal was noticed at the 2‐mm level.

### 3.2 Mesial Root and Mesial Root Canals Apical Anatomy

The average aspect ratio at the 3‐mm level above the anatomic apex of the mesial roots was 2.90 (range: 1.67–4.87). Accessory canals were identified in 28.58% (*n* = 12) of the evaluated roots. In the apical region, 33.33% (*n* = 14) of the roots had only one major foramen, followed by configurations with two (23.81%; *n* = 10), three (35.71%; *n* = 15), and four apical foramina (7.15%; *n* = 3). An independent middle mesial foramen was observed in 38.09% of cases (*n* = 16). When present, it was located mainly on the mesial wall of the root. Based on the classification by Hsu and Kim [13], the most prevalent isthmus type was Type III (52.38%; *n* = 22), followed by Type I (47.62%; *n* = 20). The average vertical distance from the mesiobuccal foramen to the anatomic apex was 0.27 mm (range: 0.00–1.16 mm), 0.37 mm (range: 0.00–1.46 mm) from the middle mesial foramen, and 0.30 mm (range: 0.00–1.02 mm) from the mesiolingual foramen. Table 2 summarises the apical anatomy of the mesial root and its associated root canals.

**TABLE 2:** Mesial root and mesial root canals apical morphology.

Sample	Root aspect ratio^a^	Accessory anatomy			Main foramen features				
		Accessory canals (*n*)	Apical foramina (*n*)	Isthmus type	Middle‐mesial independent foramen	Middle‐mesial foramen position	Vertical distance to the anatomic apex (mm)		
							Mesiobuccal	Middle‐mesial	Mesiolingual
1	2.80	0	1	III	No	—	0.43^b^		
2	3.20	3	4	I	Yes	Mesial wall	0.00	0.43	0.00
3	2.67	0	3	III	Yes	Mesial wall	0.00	1.46	0.00
4	3.00	0	3	III	Yes	Mesial wall	0.00	0.24	0.19
5	2.10	0	2	III	No	—	0.43	—	0.55
6	2.46	0	3	III	Yes	Distal wall	0.71	0.47	0.55
7	3.06	0	1	III	No	—	0.00^b^		
8	3.40	0	1	III	No	—	0.51^b^		
9	2.57	3	3	III	No	—	0.59	—	1.02
10	3.40	0	2	III	No	—	0.00	—	0.27
11	2.77	0	2	I	No	—	0.00	—	0.00
12	2.90	0	1	I	No	—	0.43^b^		
13	3.32	0	3	III	Yes	Distal wall	0.35	0.71	0.19
14	3.18	1	3	I	Yes	Mesial wall	0.63	0.24	0.39
15	2.22	0	1	III	No	—	0.35^b^		
16	2.59	0	1	III	No	—	0.66^b^		
17	3.10	2	2	III	No	—	0.00	—	0.00
18	2.44	1	3	III	Yes	Mesial wall	0.00	0.24	0.51
19	2.85	1	3	I	Yes	Distal wall	0.16	0.00	0.24
20	3.56	1	4	I	Yes	Distal wall	0.00	0.16	0.51
21	3.03	1	3	I	Yes	Mesial wall	0.00	0.20	0.19
22	3.21	0	3	III	Yes	Mesial wall	0.24	0.31	0.22
23	2.16	0	2	I	No	—	0.66	—	0.35
24	4.42	1	3	I	Yes	Distal wall	0.40	0.85	0.00
25	4.87	0	3	I	Yes	Mesial wall	0.27	0.00	0.19
26	1.67	0	1	I	No	—	1.02^b^		
27	3.40	0	2	I	No	—	0.35	—	0.16
28	2.31	0	3	I	Yes	Distal wall	0.19	0.31	0.00
29	4.78	1	2	I	No	—	0.17	—	0.31
30	2.03	0	1	I	No	—	0.15^b^		
31	2.29	0	1	III	No	—	0.00^b^		
32	2.47	0	1	III	No	—	0.44^b^		
33	2.33	3	4	III	No	—	0.13	—	0.55
34	2.35	0	1	III	No	—	0.19^b^		
35	2.91	0	2	I	No	—	0.57	—	0.85
36	4.08	0	2	I	No	—	0.24	—	0.17
37	1.84	0	1	I	No	—	0.00^b^		
38	2.10	1	2	I	No	—	1.16	—	0.11
39	2.44	0	1	III	No	—	0.59^b^		
40	3.34	0	3	I	Yes	Distal wall	0.22	0.44	0.83
41	3.11	0	3	III	Yes	Mesial wall	0.00	0.00	0.15
42	3.10	0	1	III	No	—	0.31^b^		

Abbreviations: mm, millimetres; *n*, sample size.

^a^ Measured 3‐mm coronal from the anatomic apex.

^b^ Represent cases where all canals merged at a single apical foramen.

## 4 Discussion

An accurate and thorough understanding of the three‐dimensional structure of the root canal system, including recognition of its anatomical variations, is crucial for achieving successful endodontic treatments. Failure to detect and treat all root canals can leave untreated spaces that serve as reservoirs for bacteria. These untreated or missed canals enable bacterial survival, promoting their apical migration and establishing pathways for direct communication with periapical tissues. Such conditions can initiate or sustain infections, ultimately leading to the development of persistent apical periodontitis and compromising the long‐term success of the procedure [7, 14]. A missed canal is often attributed to an operator's limited understanding or misinterpretation of the tooth's internal anatomy. Therefore, the findings of the present research highlight the crucial need for clinicians to recognise the potential for anatomic variations and the risks they pose during root canal treatment. By pursuing comprehensive anatomical knowledge and employing advanced diagnostic tools, clinicians can enhance their ability to identify and treat all canals, including complex or accessory canals like the MMC. Such a meticulous approach is essential to reduce the likelihood of untreated anatomy and improve the chances of achieving successful outcomes in both surgical and nonsurgical endodontic treatments [15, 16].

The present study confirmed the high variability and frequent presence of anatomical anomalies in the root canal configuration of the mesial root of mandibular molars. Among the 42 M analysed, only one mesial root was classified as Vertucci type VIII. The remaining samples exhibited varying degrees of complexity and were categorised according to the classification system proposed by Versiani et al. [5]. These findings are consistent with results from multiple micro‐CT studies conducted on different tooth types and across diverse populations [4, 6, 17].

The broad range of canal configurations identified in this study may be attributed to the frequent presence of canal communications, including isthmuses, which are often difficult to detect using conventional imaging techniques. The higher prevalence of a third or even fourth canal in the mesial root, such as the 8.96% proportion of three canals observed in this study, compared to the 2.30% reported by de Pablo et al. [17], may be explained by the enhanced detection capabilities of micro‐CT. Although micro‐CT is not feasible for clinical use, it was employed in the present investigation to assess the root canal morphology of mandibular molars with a MMC, as it offers more detailed and comprehensive anatomical insights than conventional methods, including clearing techniques, two‐dimensional radiography, and even cone‐beam computed tomography (CBCT). Tables 3 and 4 summarise the findings from CBCT in vivo studies and ex vivo studies using micro‐CT and clearing techniques, respectively, regarding the prevalence of MMCs in mandibular molars. Additionally, micro‐CT enables the acquisition of quantitative data on anatomical structures [76, 77].

**TABLE 3:** CBCT in vivo studies reporting prevalence of the middle mesial canal in mandibular molars.

Study (First author, year)	Region	Patients (*n*)	Male/female (*n*/*n*)	Tooth sample size (*n*)	Middle mesial canal percentage (%)	Root canal system configuration^a^
**First mandibular molar**						
Torres et al. 2015 [18]	Belgium	100	52/48	145	0.71	3‐3 (0.71%)
Caputo et al. 2016 [19]	Brazil	198	92/106	342	0.90	2‐3 (0.30%); 3‐1 (0.60%)
Silva et al. 2013 [20]	Brazil	154	70/84	234	0	—
Torres et al. 2015 [18]	Chile	170	75/95	146	3.65	3‐3 (3.65%)
Hu et al. 2019 [21]	China	496	249/247	823	10.80	N/A
Ni et al. 2018 [22]	China	646	302/344	900	1.90	3‐3 (1.90%)
Wang et al. 2010 [23]	China	558	301/257	410	2.60	3‐3 (0.20%); 3‐2 (1.20%); 3‐2‐1 (1.00%); 2‐3 (0.20%)
Zhang et al. 2011 [24]	China	211	101/110	232	0	—
Zhang et al. 2015 [25]	China	455	257/198	910	0	—
Peter Hatipoğlu et al. 2023 [26]	Croatia	163	50/113	326	0.01	—
Peter Hatipoğlu et al. 2023 [26]	Egypt	503	308/195	1006	0.01	—
Peter Hatipoğlu et al. 2023 [26]	Germany	248	122/126	496	0.15	—
Felsypremila et al. 2015 [27]	India	246	—	299	3.70	N/A
Peter Hatipoğlu et al. 2023 [26]	India	491	395/96	982	0.10	—
Kashyap et al. 2017 [28]	India	100	—	200	1.50	N/A
Madani et al. 2017 [29]	Iran	110	62/92	154	0	—
Shakeri et al. 2019 [30]	Iran	207	81/126	207	4.00	N/A
Gambarini et al. 2018 [31]	Italy	130	—	200	0	—
Plotino et al. 2013 [32]	Italy	201	80/121	117	0.85	N/A
Peter Hatipoğlu et al. 2023 [26]	Jordan	502	202/300	1004	0.02	—
Peter Hatipoğlu et al. 2023 [26]	Kazakhstan	499	263/236	998	0.05	—
Peter Hatipoğlu et al. 2023 [26]	Libya	503	189/314	1006	0.23	—
Kim et al. 2013 [33]	Korea	976	460/516	1952	0.35	2‐3‐2 (0.21%); 3‐2 (0.14%)
Deng et al. 2018 [34]	Malaysia	208	92/116	301	0.80	N/A
Peter Hatipoğlu et al. 2023 [26]	Malaysia	519	198/321	1038	0.02	—
Peter Hatipoğlu et al. 2023 [26]	Pakistan	160	78/82	320	0.08	—
Nazeer et al. 2019 [35]	Pakistan	78	—	142	1.40	N/A
Shah et al. 2019 [36]	Pakistan	120	—	120	0.80	3‐3 (0.80%)
Peter Hatipoğlu et al. 2023 [26]	Poland	540	315/225	1080	0.01	—
Peter Hatipoğlu et al. 2023 [26]	Portugal	608	305/303	1216	0.04	—
Peter Hatipoğlu et al. 2023 [26]	Saudi Arabia	254	168/86	508	0.13	—
Srivastava et al. 2018 [37]	Saudi Arabia	82	43/39	143	18.20	N/A
Peter Hatipoğlu et al. 2023 [26]	South Africa	393	176/217	786	0.13	—
Pérez‐Heredia et al. 2017 [38]	Spain	112	56/56	121	8.40	3‐2 (5.90%); 2‐3‐1 (1.70%); 3‐2‐3 (0.80%)
Huang et al. 2010 [39]	Taiwan	151	76/75	237	0	—
Ahmetoğlu et al. 2014 [40]	Turkey	152	79/73	173	0	—
Celikten et al. 2016 [41]	Turkey	272	—	384	0	—
Demirbuga et al. 2013 [42]	Turkey	605	268/337	823	0	—
Peter Hatipoğlu et al. 2023 [26]	Turkey	668	316/352	1336	0.08	—
Miloglu et al. 2013 [43]	Turkey	307	120/187	533	2.30	3‐3 (1.30%); 3‐2 (0.60%); 2‐3 (0.40%)
Nur et al. 2014 [44]	Turkey	850	429/421	966	0.20	3‐3 (0.20%)
Akbarzadeh et al. 2017 [45]	USA	210	134/76	210	14.70	N/A
Pham et al. 2019 [46]	Vietnam	166	94/72	332	0	—
Peter Hatipoğlu et al. 2023 [26]	Yemen	253	108/145	506	0.02	—
**Second mandibular molar**						
Torres et al. 2015 [18]	Belgium	100	52/48	112	2.13	3‐3 (2.13%)
Silva et al. 2013 [20]	Brazil	154	70/84	226	0	—
Torres et al. 2015 [18]	Chile	170	75/95	112	2.06	3‐3 (2.06%)
Zhang et al. 2011 [24]	China	211	101/110	157	0	—
Felsypremila et al. 2015 [27]	India	246	—	322	1.00	N/A
Kashyap et al. 2017 [28]	India	100	—	200	0	—
Pawar et al. 2017 [47]	India	532	—	983	0	—
Janani et al. 2018 [48]	Iran	231	94/137	384	0	—
Madani et al. 2017 [29]	Iran	110	62/85	147	0	—
Shakeri et al. 2019 [30]	Iran	235	95/140	235	3.00	N/A
Plotino et al. 2013 [32]	Italy	201	80/121	161	0	—
Kim et al. 2015 [49]	Korea	960	441/519	1920	0	—
Pérez‐Heredia et al. 2017 [38]	Spain	112	56/56	121	3.00	3‐1 (1.0%); 3‐2 (2.0%)
Ahmetoğlu et al. 2014 [40]	Turkey	152	79/73	235	0	—
Celikten et al. 2016 [41]	Turkey	272	—	421	0	—
Demirbuga et al. 2013 [42]	Turkey	605	268/337	925	0	—
Nur et al. 2014 [44]	Turkey	850	429/421	1165	0	—
**Third mandibular molar**						
Somasundaram et al. 2017 [50]	India	171	103/68	144	0	—

Abbreviations: *n*, sample size; N/A, not available.

^a^ Proportions as reported in the original studies.

**TABLE 4:** Ex vivo micro‐CT and clearing studies reporting prevalence of the middle mesial canal in mandibular molars.

Study (Author, year)	Methodology	Region	Tooth sample size (*n*)	Middle mesial canal percentage (%)	Root canal system configuration^a^
**First mandibular molar**					
Marceliano‐Alves et al. 2019 [8]	Micro‐CT	Brazil	104	7.70	3‐3 (7.70%)
Versiani et al. 2016 [5]	Micro‐CT	Brazil	136	22.10	3‐3 (2.21%); 1‐3 (0.74%); 1‐2‐3 (1.47%); 2‐3 (0.74%); 3‐1‐3 (0.74%); 2‐3‐2‐1‐2‐3 (1.47%); 2‐3‐2‐3 (0.74%); 2‐3‐2‐1‐2 (0.74%); 3‐2‐1 (0.74%); 2‐3‐2 (0.74%); 3‐2‐3 (2.21%); 3‐1‐2‐3 (0.74%); 2‐3‐4‐2 (0.74%) 2‐3‐4‐3‐2‐3 (1.47%); 2‐3‐1 (0.74%); 3‐2‐3‐2‐1 (0.74%); 1‐3‐2‐1 (0.74%); 3‐2‐3‐2‐3 (0.74%); 3‐2‐3‐2 (0.74%); 3‐2‐3‐4‐2 (0.74%); 2‐3‐2 (0.74%); 3‐2‐3‐2 (0.74%); 2‐3‐4‐3‐4 (0.74%)
Gulabivala et al. 2001 [51]	Clearing	Burma	139	8.60^b^	3‐1 (1.90%); 2‐3 (6.70%)
Moe et al. 2017 [1]	Micro‐CT	Burma	75	18.70	N/A
Fu et al. 2022 [9]	Micro‐CT	China	136	0	—
Saber et al. 2023 [4]	Micro‐CT	Egypt	96	51.00	3‐1 (3.13%); 3‐2 (3.13%); 2‐3 (3.13%); 1‐2‐3 (1.04%); 3‐2‐1 (5.21%); 2‐3‐2 (4.17%); 3‐2‐3 (1.04%); 2‐3‐1 (3.13%); 2‐4‐3 (1.04%); 3‐4‐3 (1.04%); 4‐3‐2 (2.08%); 4‐2‐3 (1.04%); 1‐3‐2‐1 (3.13%); 2‐3‐2‐1 (5.21%); 2‐3‐2‐3 (2.08%); 3‐2‐3‐2 (2.08%); 3‐2‐3‐4 (1.04%); 3‐2‐1‐2 (2.08%); 3‐2‐3‐4 (1.04%); 3‐4‐3‐2 (1.04%); 2‐3‐2‐3‐2 (1.04%); 1‐2‐3‐2‐1 (2.08%); 2‐3‐2‐1‐2 (1.04%); 3‐4‐5‐3‐1 (1.04%); 2‐3‐4‐3‐2 (1.04%); 2‐3‐2‐1‐3 (1.04%); 2‐3‐2‐1‐2 (1.04%); 2‐3‐2‐1‐2‐1 (1.04%); 2‐3‐2‐1‐3‐2 (1.04%); 1‐2‐3‐2‐3‐2 (1.04%); 3‐1‐2‐1‐2‐1 (1.04%); 3‐2‐3‐2‐3‐2‐3‐2 (1.04%); 2‐3‐4‐2‐1‐2‐3 (1.04%)
Wolf et al. 2016 [10]	Micro‐CT	Egypt	118	7.50	2‐2‐3 (3.40%); 2‐3‐3 (1.70%); 2‐3‐1 (0.80%); 2‐3‐2 (0.80%); 3‐3‐1 (0.80%)
Chourasia et al. 2012 [52]	Clearing	India	150	0	—
Singh et al. 2020 [53]	Clearing	India	100	0	—
Akhlaghi et al. 2017 [54]	Clearing	Iran	150	0	—
Shahi et al. 2008 [55]	Clearing	Iran	209	0	—
Al‐Qudah et al. 2009 [56]	Clearing	Jordan	330	6.00	3‐3 (0.30%); 2‐3 (1.50%); 2‐3‐1 (0.60%); 2‐3‐2 (0.90%); 3‐1 (0.60%); 3‐2 (1.20%); 3‐2‐1 (0.30%); 3‐2‐3 (0.60%)
Asijavičienė et al. 2020 [57]	Micro‐CT	Lithuania	60	1.85	N/A
Wasti et al. 2001 [58]	Clearing	Pakistan	30	3.30	3‐3 (3.30%)
Mukhaimer et al. 2014 [59]	CBCT	Palestine	320	3.10	3‐2 (2.50%); 3‐2‐1 (0.60%)
Tomaszewska et al. 2018 [12]	Micro‐CT	Poland	108	0.50	3‐3 (0.50%)
Navarro et al. 2007 [60]	CBCT	Spain	27	14.80	N/A
Peiris et al. 2007 [61]	Clearing	Sri Lanka	100	9.00	3‐3 (1.00%); 2‐3, 1‐2‐3, 3‐2‐1 (8.00%)
Ahmed et al. 2007 [62]	Clearing	Sudan	100	7.00	3‐3 (2.00%); 3‐1 (5.00%)
Chen et al. 2009 [63]	Clearing	Taiwan	183	5.46	2‐3 (5.46%)
Madjapa et al. 2018 [64]	Clearing	Tanzania	146	0	—
Gulabivala et al. 2002 [65]	Clearing	Thailand	118	4.60	3‐3 (1.90%); 3‐2 (0.90%); 3‐4 (0.90%); 2‐3 (0.90%)
Keleş et al. 2020 [3]	Micro‐CT	Turkey	30	33.30	N/A
Keleş et al. 2018 [66]	Micro‐CT	Turkey	269	15.00	N/A
Şallı & Egil., 2021 [6]	Micro‐CT	Turkey	40	25.00	2‐3 (2.50%); 2‐3‐1 (2.50%); 3‐2‐1 (2.50%); 1‐3‐2‐3 (2.50%); 1‐2‐3‐1 (2.50%); 2‐1‐2‐3 (2.50%); 1‐2‐3‐1 (2.50%); 2‐3‐2‐3 (2.50%); 1‐2‐3‐2‐3 (2.50%); 1‐2‐1‐2‐1 (2.50%)
Sert et al. 2011 [67]	Clearing	Turkey	411	2.90^b^	3‐3 (0.73%); 1‐2‐3‐2 (1.21%); 3‐1 (0.24%); 3‐1‐2 (0.24%); 2‐3‐4‐2 (0.24%); 3‐1‐2‐1‐2 (0.24%)
Sert et al. 2004 [68]	Clearing	Turkey	200	4.00	3‐3 (1.50%); 1‐2‐3‐2 (2.50%)
Versiani et al. 2016 [5]	Micro‐CT	Turkey	122	14.80	2‐3‐2‐1 (1.64%); 3‐2‐1 (0.82%); 3‐2‐3‐2‐1 (0.82%); 3‐1‐2‐1 (0.82%); 3‐1‐2‐3 (0.82%); 3‐2‐1‐3 (0.82%); 3‐2‐3‐2‐3‐2 (0.82%); 3‐2‐4 (0.82%); 3‐1‐3 (0.82%); 3‐2‐3‐2‐3 (1.64%); 3‐2‐4‐2 (0.82%); 3‐2‐1 (1.64%); 3‐2‐3‐1 (0.82%); 3‐2‐3 (0.82%); 2‐3‐2‐1‐2 (0.82%)
Rwenyonyi et al. 2009 [69]	Clearing	Uganda	224	0	—
Harris et al. 2013 [70]	Micro‐CT	USA	22	36.00	2‐3‐1‐2 (4.55%); 2‐3‐1 (4.55%); 2‐3‐2‐1 (4.55%); 2‐3‐2 (4.55%); 2‐4‐3‐2 (4.55%); 3‐2‐1 (9.10%); 3‐4‐3‐2‐1 (4.55%)
**Second mandibular molar**					
Gulabivala et al. 2001 [51]	Clearing	Burma	134	0	—
Wolf et al. 2017 [16]	Micro‐CT	Egypt	93	10.80	2‐3‐2 (4.30%); 2‐3‐1 (3.20%); 2‐2‐3 (1.10%); 3‐2‐1 (1.10%); 3‐3‐2 (1.10%)
Neelakantan et al. 2010 [71]	Clearing	India	345	3.20^b^	3‐1 (2.00%); 3‐2 (1.20%)
Singh et al. 2020 [53]	Clearing	India	100	0	—
Jahromi et al. 2013 [72]	Clearing	Iran	100	0	—
Rahimi et al. 2008 [73]	Clearing	Iran	139	0.80	3‐3 (0.80%)
Al‐Qudah et al. 2009 [56]	Clearing	Jordan	355	2.80	3‐3 (0.30%); 2‐3 (0.60%); 2‐3‐1 (0.30%); 2‐3‐2 (1.00%); 3‐2 (0.30%); 3‐2‐1 (0.30%)
Tomaszewska et al. 2018 [12]	Micro‐CT	Poland	120	0.10	3‐3 (0.10%)
Peiris et al. 2007 [61]	Clearing	Sri Lanka	100	2.00	2‐3, 1‐2‐3, 3‐2‐1 (2.00%)
Ahmed et al. 2007 [62]	Clearing	Sudan	100	0	—
Madjapa et al. 2018 [64]	Clearing	Tanzania	85	0	—
Gulabivala et al. 2002 [65]	Clearing	Thailand	60	3.80	3‐2 (1.90%); 2‐3 (1.90%)
Sert et al. 2011 [67]	Clearing	Turkey	318	1.88^b^	1‐2‐3‐2 (1.88%)
Sert et al. 2004 [68]	Clearing	Turkey	200	3.50	1‐2‐3‐2 (3.50%)
Rwenyonyi et al. 2009 [69]	Clearing	Uganda	223	0	—
Barsness et al. 2015 [11]	Micro‐CT	USA	18	16.70	N/A
**Third mandibular molar**					
Gulabivala et al. 2001 [51]	Clearing	Burma	58	0	—
Singh et al. 2020 [53]	Clearing	India	100	0	—
Kuzekanani et al. 2012 [74]	Clearing	Iran	150	0	—
Ahmad et al. 2016 [75]	Clearing	Jordan	70	0^b^	—
Al‐Qudah et al. 2023 [56]	Clearing	Jordan	639	0.78^b^	3‐3 (0.78%)
Tomaszewska et al. 2018 [12]	Micro‐CT	Poland	146	0.10	3‐3 (0.1%)
Sert et al. 2011 [67]	Clearing	Turkey	256	0^b^	—
Gulabivala et al. 2002 [65]	Clearing	Thailand	173	0^b^	—

Abbreviations: *n*, sample size; N/A, not available.

^a^ Proportions as reported in the original studies.

^b^ Only teeth with two separate roots.

Assessing dentine thickness is critical, as excessive dentine removal during endodontic procedures can lead to root fractures or perforations [3, 70]. At the axial level below the furcation, the average periradicular dentine thickness associated with the MMC was 1.02 mm (range: 0.68–1.40 mm). This finding aligns with the dentine thickness values reported by Saber et al. [4], who observed mean measurements of 1.05 ± 0.4 mm toward the mesial wall and 0.99 ± 0.38 mm toward the distal wall. Similarly, Versiani et al. [5] reported dentine thicknesses ranging from 0.80 to 2.20 mm on the furcation side relative to the MMC orifice. The frequent presence of dentine thickness values below 1 mm around the MMC highlights the need for conservative shaping strategies, including careful taper selection, limited access enlargement, and caution during ultrasonic troughing, to minimise the risk of strip perforation or structural weakening of the root.

The aspect ratio has been recognised as an indicator of anatomical complexity in root canals and has been explored in previous endodontic studies [78]. Ginjeira et al. [79] reported that a higher root aspect ratio was associated with increased internal anatomical complexity in mandibular molars. Specifically, mesial roots with an aspect ratio ≥ 2.75 exhibited a significantly greater number of main canals at all axial levels, and the two independent MMCs identified in their study were both found in roots with higher aspect ratios. These findings are consistent with those of the present study, in which the average aspect ratio at the 3‐mm level above the anatomic apex of the mesial roots was 2.90.

As our study focused exclusively on mandibular molars with an MMC, isthmus configurations, which were categorised according to the classification proposed by Hsu and Kim in 1997 [13], could only be classified as Type I (three canals with no communication), Type III (three canals with a definite connection), or Type V (a continuous corridor throughout the section). Type III was the most frequently observed isthmus type, while no specimens were classified as Type V. The high prevalence of isthmuses observed aligns with previous reports that describe prevalence rates ranging from 54% to 89%. In the present study, the prevalence of isthmuses was 52.38%, although the distribution of isthmus types differed slightly from earlier reports [1, 8, 57, 70].

Regarding the vertical distance from the main foramen to the anatomic apex, all canals demonstrated mean values below 1 mm. This is in agreement with the findings of Ginjeira et al. [79], confirming that the majority of foraminal exits are located within the apical 3 mm. These results support the recommendation by Setzer and Kratchman [80], which proposes 3 mm as the clinical guideline for root‐end resection during surgical procedures, an approach shown to eliminate 98% of apical ramifications and 93% of lateral canals [81].

A comprehensive understanding of the complex and variable root canal anatomy of mandibular molars with MMCs is essential for achieving successful endodontic outcomes. Such anatomical insight is critical for accurate diagnosis and the effective performance of both surgical and nonsurgical endodontic procedures. Clinicians should carefully consider anatomical complexity before initiating treatment to reduce the risk of iatrogenic complications during shaping and cleaning. In this context, micro‐CT proves to be an invaluable tool, offering high‐resolution, three‐dimensional imaging. From a clinical perspective, the quantitative anatomical data provided by micro‐CT—particularly regarding dentine thickness, aspect ratio, and isthmus prevalence—may inform decision‐making related to instrument selection, shaping strategy, and the extent of apical resection or retropreparation during surgical endodontics.

The current study presents some limitations that should be acknowledged. Due to the anonymised nature of the samples, it was not possible to correlate anatomical findings with demographic variables such as age or sex, which constitutes a methodological constraint and further limits generalisability. Consequently, potential differences in the prevalence or configuration of the MMC related to sex or age‐associated changes in root canal morphology could not be explored. Additionally, the exclusive use of tooth‐bank specimens introduces inherent selection bias, as extracted teeth may not accurately represent the anatomical variability of the general population, thereby further limiting the external validity of the findings. Moreover, the measurement procedures, although conducted with high‐resolution micro‐CT, are still subject to potential measurement variability related to image segmentation, voxel size limitations, and operator‐dependent interpretation, which should be considered when extrapolating these results. These combined factors may restrict the extent to which the present findings can be generalised to broader or demographically diverse patient subgroups.

Nevertheless, the study also demonstrates notable strengths. The inclusion of 42 mandibular molars with an MMC constitutes a comparatively large sample size within the existing micro‐CT literature, particularly given the uncommon nature of this anatomical feature. The use of high‐resolution micro‐CT provided detailed, three‐dimensional, nondestructive imaging, allowing for an in‐depth morphological characterisation that included quantitative assessment of dentine thickness, isthmus types, apical foramina, and complex canal configurations, surpassing the limitations of conventional methods. Additionally, the findings were interpreted within the context of an extensive and up‐to‐date global body of literature, enabling meaningful comparison with previous ex vivo and in vivo studies across different populations. To enhance the clinical applicability of future research, studies should aim to incorporate larger, demographically characterised samples. The integration of patient‐specific data, such as age, sex, and ethnicity, could enable the investigation of anatomical variability across populations. Furthermore, complementing ex vivo findings with in vivo imaging approaches like CBCT may help validate and contextualise results within clinical practice. These efforts will be relevant in refining diagnostic strategies and improving the predictability and outcomes of endodontic treatments.

## 5 Conclusions

This study showed that mesial roots of mandibular molars with a MMC present substantial anatomical variability in both root canal configuration and apical morphology. The confluent configuration was the most frequent, while a fully independent MMC was rare. The MMC consistently exhibited the lowest periradicular dentine thickness, often below 1 mm at 2 mm below the furcation, posing an increased risk during canal preparation. The mean aspect ratio of 2.90 at 3 mm above the anatomic apex reflected marked anatomical complexity, frequently associated with accessory canals, multiple foramina, and isthmus formations, most commonly Type III. These features emphasise the clinical relevance of recognising the potential presence and variability of the MMC. Awareness of these anatomical nuances is essential to guide endodontic procedures, minimise iatrogenic complications, and improve treatment outcomes.

## Conflicts of Interest

The authors declare no conflicts of interest.

## Data Availability

Data sharing not applicable to this article as no datasets were generated or analysed during the current study.
